# Supplementary material for: Non-linear changes in modelled terrestrial ecosystems subjected to perturbations
Source: Sci Rep. 2020 Aug 20;10:14051. doi: 10.1038/s41598-020-70960-9 (PMC7441154; doi:10.1038/s41598-020-70960-9)
Supplement: Supplementary file 1 — Supplementary information. [file 41598_2020_70960_MOESM1_ESM.docx]

Supplementary Material for ‘Non-linear changes in modelled terrestrial ecosystems subjected to perturbations’

Tim Newbold^1,2,†,*^, Derek P. Tittensor^1,3,†^, Michael B. J. Harfoot^1,†^, Jörn P. W. Scharlemann^1,4^, Drew W. Purves^5,‡^

^1^UN Environment Programme World Conservation Monitoring Centre, 219 Huntingdon Road, Cambridge CB3 0DL, United Kingdom.

^2^Centre for Biodiversity and Environment Research, Department of Genetics, Evolution and Environment, University College London, Gower Street, London WC1E 6BT, United Kingdom.

^3^Biology Department, Dalhousie University, 1355 Oxford Street, Halifax, Nova Scotia B3H 4R2, Canada.

^4^School of Life Sciences, University of Sussex, Brighton BN1 9QG, United Kingdom.

^5^Computational Science Laboratory, Microsoft Research, Cambridge CB1 2FB, United Kingdom

^†^These authors contributed equally.

^‡^Current address: DeepMind, 6 Pancras Square, London, N1C 4AG

*Corresponding author: Tim Newbold ([t.newbold@ucl.ac.uk](mailto:t.newbold@ucl.ac.uk))

# Appendix 1: Supplementary Figures


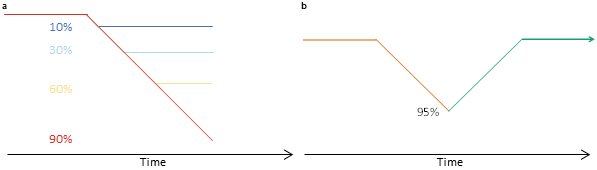


**Figure S1.** The temporal configuration of simulations of human impact. The first set of simulations (**a**), which involved gradual increase in human removal of net primary production at a constant rate but for different lengths of time, was used for most results presented. Exemplar levels of impact are shown here; for the real simulations, all levels of impact at increments of 10% between 0% and 90% were simulated. The second set of simulations (**b**), which involved gradual increase to 95% human removal of net primary production and then gradual decrease back to zero impact, was used to test for irreversibility and hysteresis in ecosystem responses.


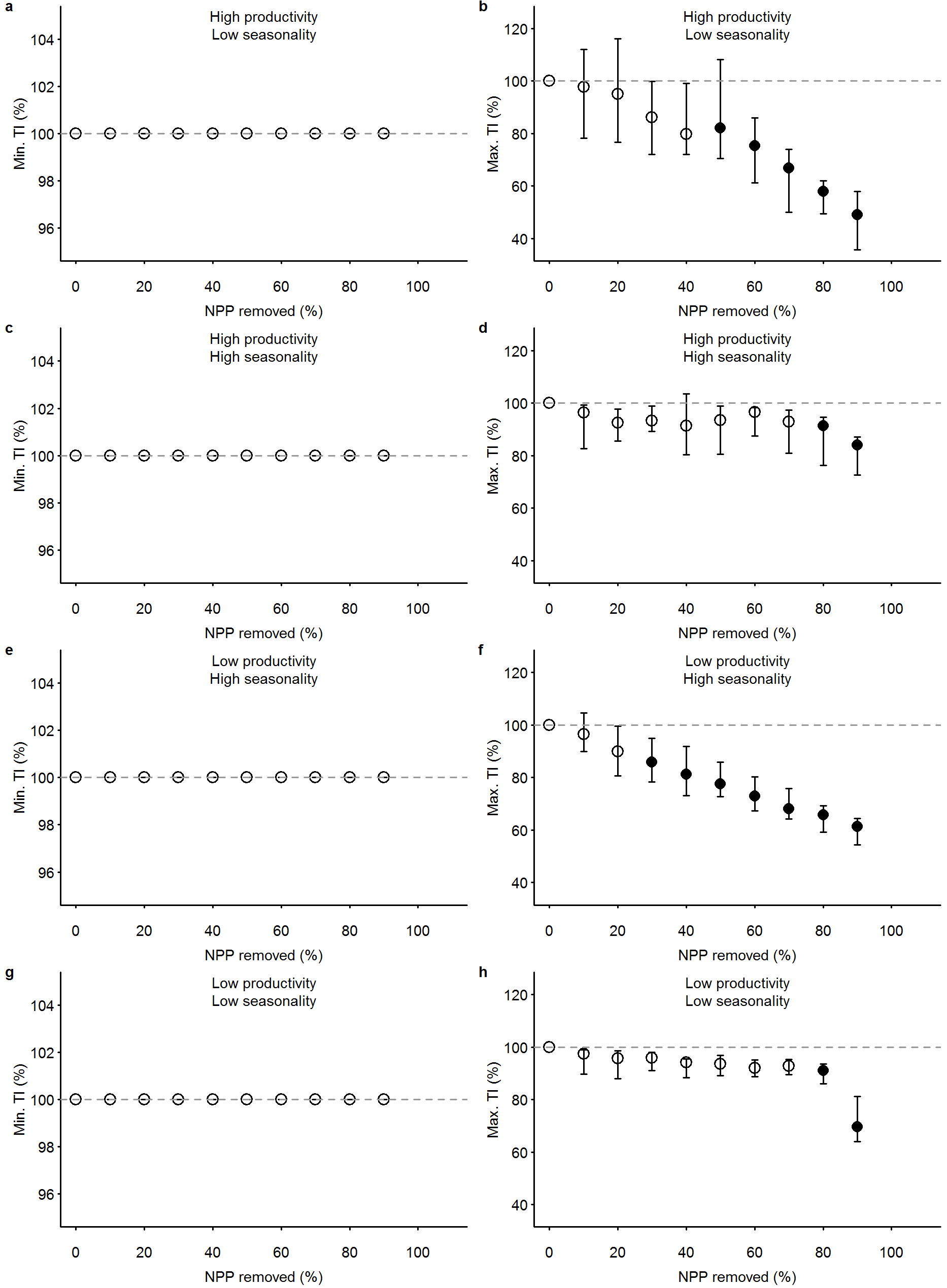


**Figure S2.** Impacts, relative to an undisturbed state, of removal of net primary production (NPP) on the minimum (**a**, **c**, **e**, **g**) and maximum (**b**, **d**, **f**, **h**) trophic indices (TI) of heterotroph organisms in four ecosystems: (**a**-**b**) high productivity, aseasonal (Uganda); (**c**-**d**) relatively high productivity, highly seasonal (France); (**e**-**f**) relatively low productivity, seasonal (Gobi Desert, China); and (**g**-**h**) low productivity, aseasonal (Libyan Desert). Ecosystems were subjected to levels of NPP removal that increased by 1% per year for between 0 and 90 years, resulting in eventual levels of NPP removal ranging between 0 and 90%. Values shown here are the ecosystem properties after a total of 100 years of impact. Error bars show ±95% confidence intervals; open points show levels of impact for which at least one of the time series did not show a significant negative trend (according to a Mann-Kendall test, run using the ‘Kendall’ package Version 2.2 in R Version 3.3.3, P < 0.05).


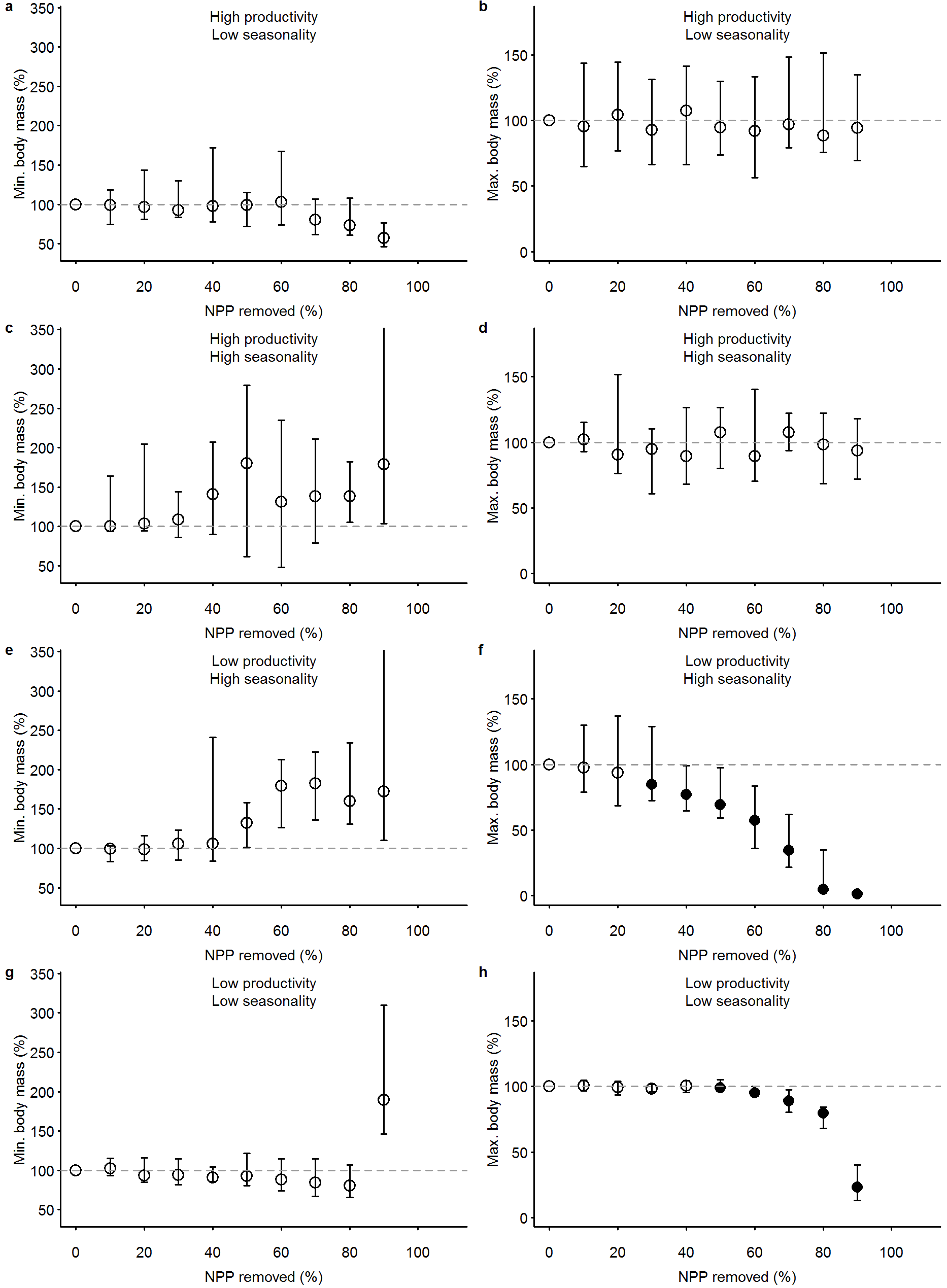


**Figure S3.** Impacts, relative to an undisturbed state, of removal of net primary production (NPP) on the minimum (**a**, **c**, **e**, **g**) and maximum (**b**, **d**, **f**, **h**) body masses of heterotroph organisms in four ecosystems: (a-b) high productivity, aseasonal (Uganda); (**c**-**d**) relatively high productivity, highly seasonal (France); (**e**-**f**) relatively low productivity, seasonal (Gobi Desert, China); and (**g**-**h**) low productivity, aseasonal (Libyan Desert). Simulations and plotting conventions are as in Figure S2.

**
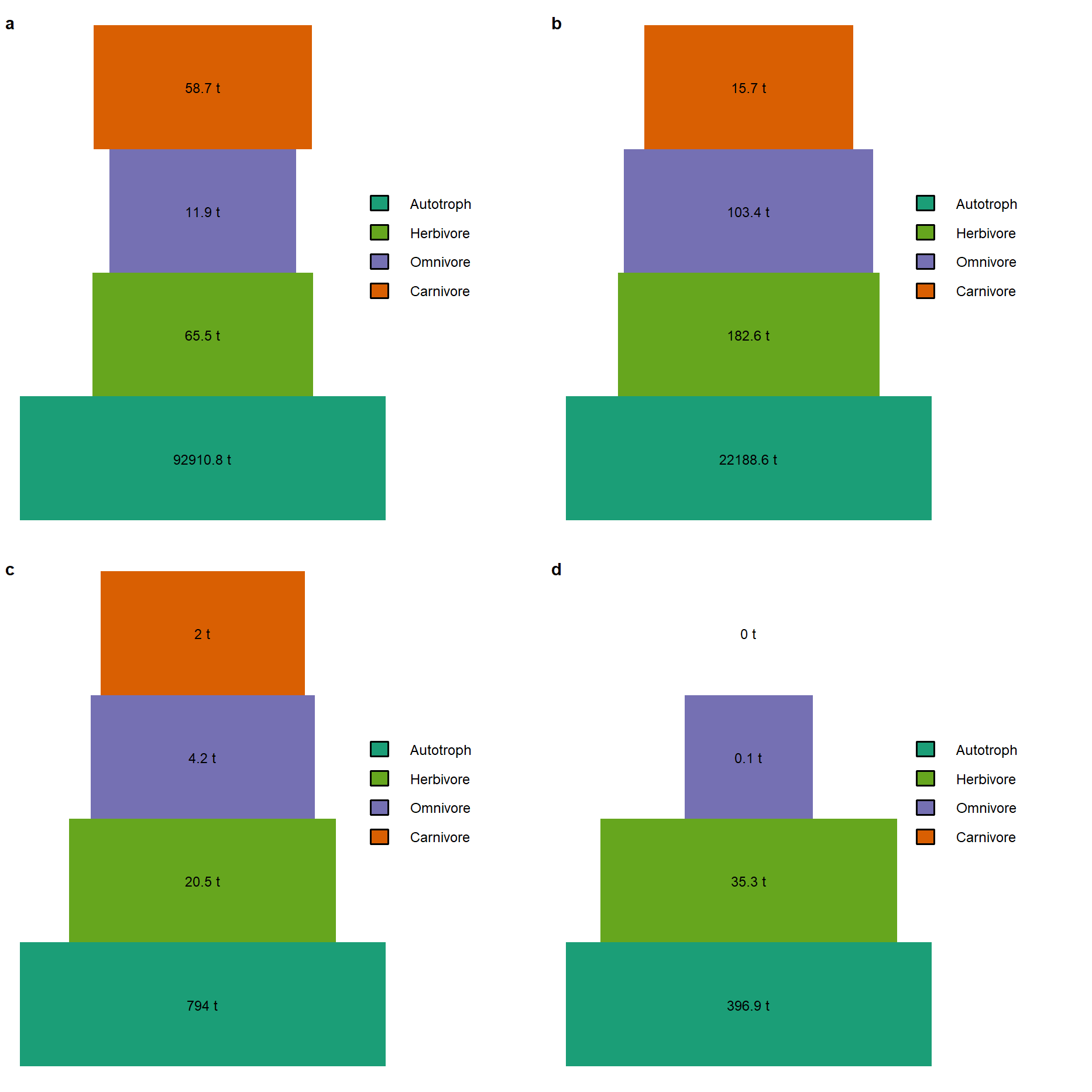
**

**Figure S4**. Trophic structure of four simulated ecosystems under natural conditions with no human removal of vegetation. Shown are biomass pyramids where the bar width is proportional to (log_e_) total biomass of each trophic level: autotrophs, herbivores, omnivores and carnivores. Numbers within the bars refer to total biomasses in metric tonnes (t). The four ecosystems are: (**a**) high productivity, aseasonal (Uganda); (**b**) relatively high productivity, highly seasonal (France); (**c**) relatively low productivity, seasonal (Gobi Desert, China); and (**d**) low productivity, aseasonal (Libyan Desert).


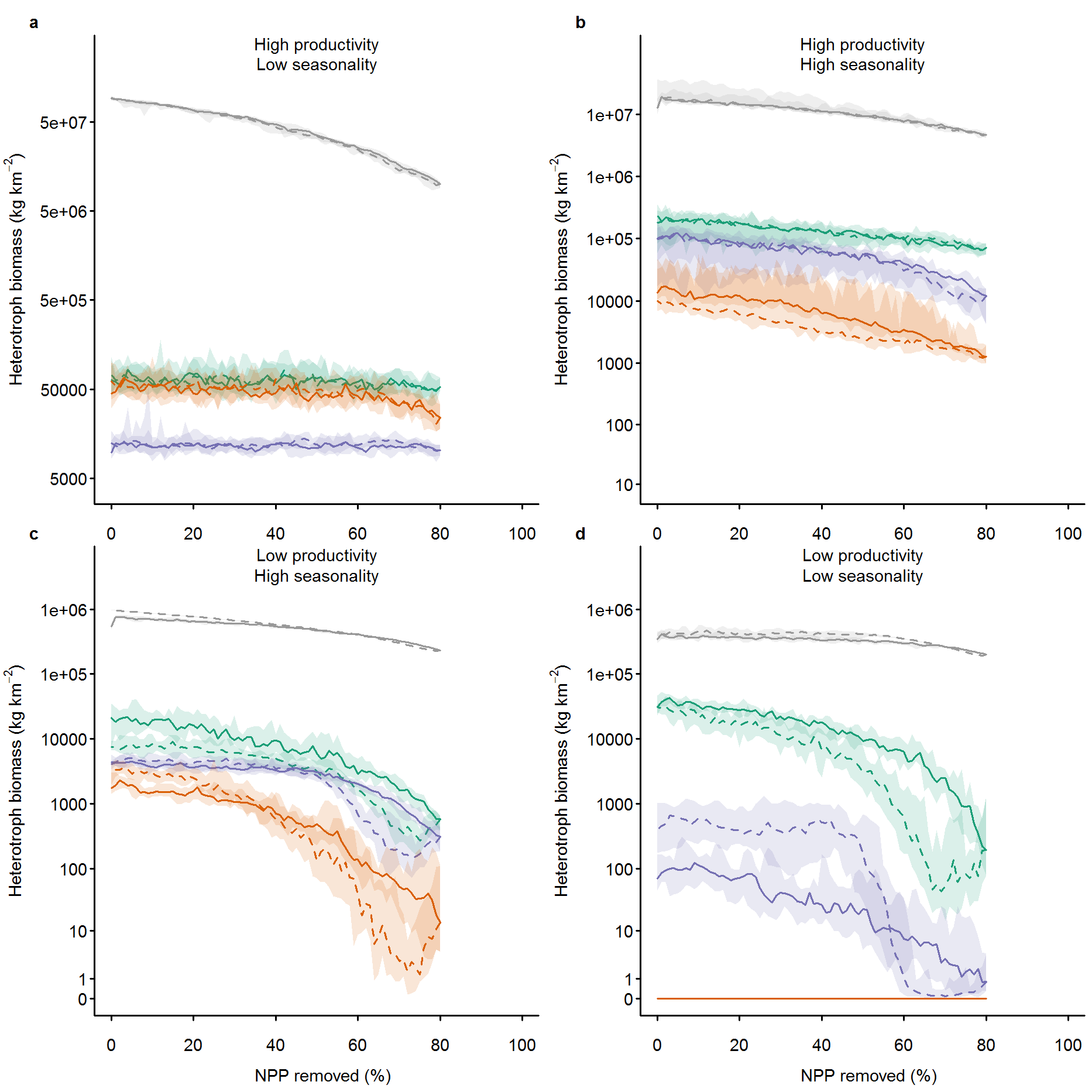


**Figure S5.** Irreversibility of change in total ecosystem biomass after gradually increasing human impact to 80% removal of net primary production (NPP) (solid lines), followed by gradual release (dotted lines) for different trophic levels ‒ autotrophs (grey), herbivores (green), omnivores (purple) and carnivores (red) ‒ in four ecosystems: (**a**) high productivity, aseasonal (Uganda); (**b**) relatively high productivity, highly seasonal (France); (**c**) relatively low productivity, seasonal (Gobi Desert, China); and (**d**) low productivity, aseasonal (Libyan Desert). Shading shows 95% confidence intervals.


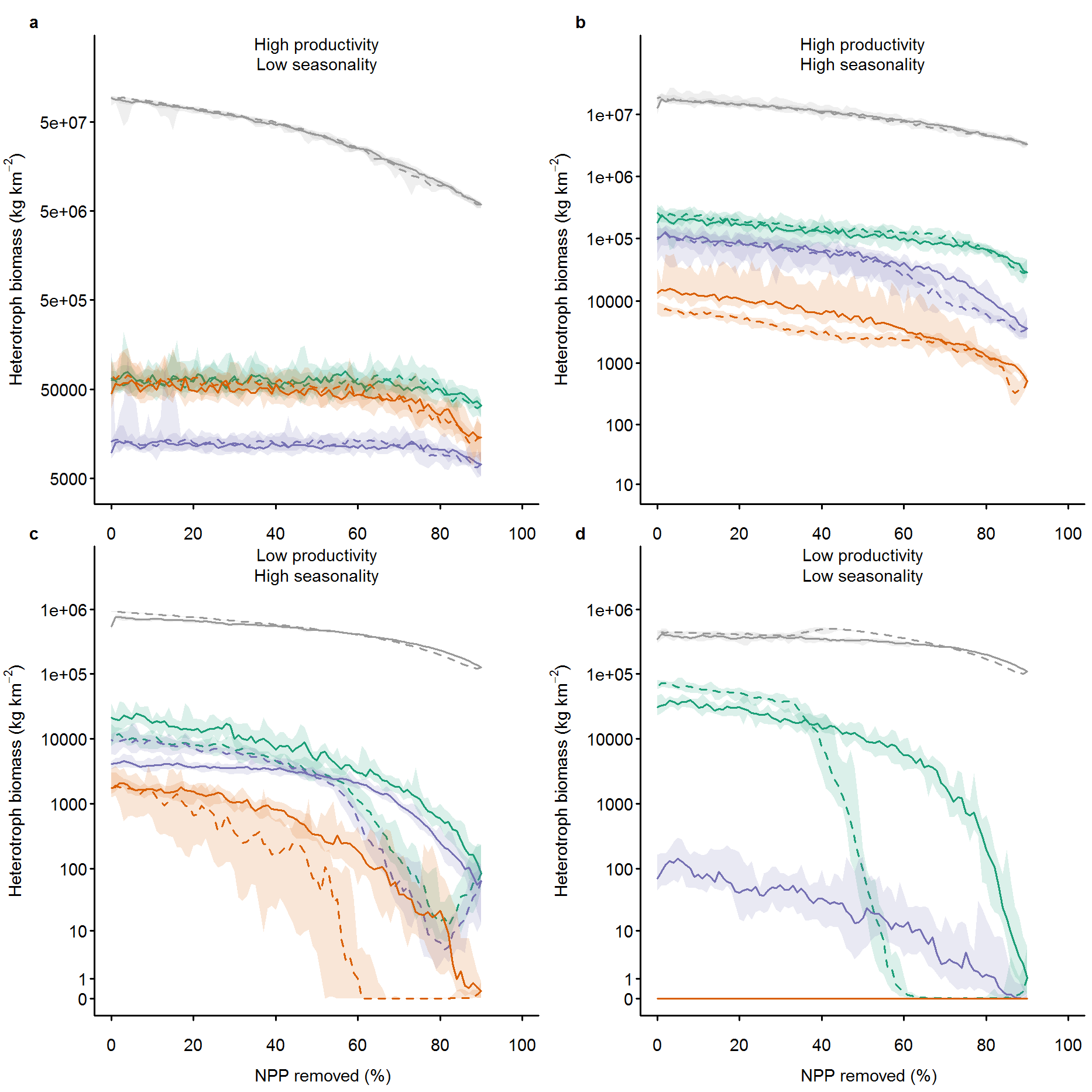


**Figure S6.** Irreversibility of change in total ecosystem biomass after gradually increasing human impact to 90% removal of net primary production (NPP) (solid lines), followed by gradual release (dotted lines) for different trophic levels ‒ autotrophs (grey), herbivores (green), omnivores (purple) and carnivores (red) ‒ in four ecosystems: (**a**) high productivity, aseasonal (Uganda); (**b**) relatively high productivity, highly seasonal (France); (**c**) relatively low productivity, seasonal (Gobi Desert, China); and (**d**) low productivity, aseasonal (Libyan Desert). Shading shows 95% confidence intervals.

# Appendix 2: Setup of regime shifts simulations

## Introduction

This document explains how to set up the simulations used in the manuscript testing for regime shifts in ecosystems subjected to human extraction of vegetation. This manuscript is currently in review. If you need any help getting the simulations running, please contact the corresponding author, Tim Newbold (t.newbold@ucl.ac.uk).

## Input data

All of the input data are contained within the repository or are pulled directly from Microsoft's FetchClimate app.

## Initialisation basics

The model is initialised through a set of CSV files (found in the folder input\Model setup). The files that need to be changed to set up the regime-shift simulations are as follows:

1. EcosystemModelInitialisation.csv - this contains basic information about the simulation.

2. ReadModelState.csv - this specifies the location of the files containing the model state from initialisation runs

3. Scenarios.csv - this specifies the details of the impact scenario

Most of the parameters are already set to the right values. Those that need to be changed will be explained in the following section.

## Setting up the impact simulations

### Setting the locations

All simulations were run on 3 × 3 grids of 1-degree cells. The grid is specified in EcosystemModelInitialisation.csv, using the parameters Bottom Latitude, Top Latitude, Leftmost Latitude, Rightmost Latitude. The latter two were misnamed and actually refer to longitudes.

The full set of parameters for the four locations simulated in the manuscript is as follows:

Target Longitude | Target Latitude | Grid Leftmost Longitude | Grid Rightmost Longitude | Grid Bottom Latitude | Grid Top Latitude

--- | --- | --- | --- | --- | ---

33 | 2 | 31.5 | 34.5 | 0.5 | 3.5

4 | 48 | 2.5 | 5.5 | 46.5 | 49.5

91 | 38 | 89.5 | 92.5 | 36.5 | 39.5

13 | 30 | 11.5 | 14.5 | 28.5 | 31.5

### Initialisation simulations

All of the initialisation simulations followed the same setup, although two separate sets of initialisation simulations were run for each of the two impact protocols.

In EcosystemModelInitialisation.csv, apart from the specification of the grid, the following parameters need to be set as follows (where there is nothing after the comma, this should be blank):

- Length of simulation (years), 1000

- Burn-in (years), 0

- Impact duration (years), 0

- Recovery duration (years), 0

- Read State,

- Output Model State Timesteps,11999

In Scenarios.csv, there should be one line under the header row as follows:

label | npp | temperature | harvesting | simulation number

--- | --- | --- | --- | ---

NI | no 0.0 0 | no 0.0 | no 0.0 | 10

### Impact simulations - 0. Reading model state

For both impact protocols, ReadModelState.csv needs to contain a list of the ModelState[x].nc files produced by the initialisation simulations, in the Filename column. Note that Path column is redundant; the model assumes that the state files are found in the folder input\ModelStates (you will need to copy your state files to this location).

### Impact simulations - 1. Press-and-release protocol

This is the protocol used in the main set of simulations presented in the manuscript, and illustrated in Figure S1A.

In EcosystemModelInitialisation.csv, apart from the specification of the grid, the following parameters need to be set as follows:

- Length of simulation (years), 210

- Burn-in (years), 10

- Impact duration (years), 100

- Recovery duration (years), 0

- Read State,ReadModelState.csv

- Output Model State Timesteps,no

In Scenarios.csv, there should be ten lines as follows:

label | npp | temperature | harvesting | simulation number

--- | --- | --- | --- | ---

NI | no 0.0 0 | no 0.0 | no 0.0 | 10

NPPTECR1 | tempescalating-const-rate-duration 0.01 10 | no 0.0 | no 0.0 | 10

NPPTECR2 | tempescalating-const-rate-duration 0.01 20 | no 0.0 | no 0.0 | 10

NPPTECR3 | tempescalating-const-rate-duration 0.01 30 | no 0.0 | no 0.0 | 10

NPPTECR4 | tempescalating-const-rate-duration 0.01 40 | no 0.0 | no 0.0 | 10

NPPTECR5 | tempescalating-const-rate-duration 0.01 50 | no 0.0 | no 0.0 | 10

NPPTECR6 | tempescalating-const-rate-duration 0.01 60 | no 0.0 | no 0.0 | 10

NPPTECR7 | tempescalating-const-rate-duration 0.01 70 | no 0.0 | no 0.0 | 10

NPPTECR8 | tempescalating-const-rate-duration 0.01 80 | no 0.0 | no 0.0 | 10

NPPTECR9 | tempescalating-const-rate-duration 0.01 90 | no 0.0 | no 0.0 | 10

### Impact simulations - 2. Escalate-and-decline protocol

This is the protocol used in the manuscript to test the reversibility of impacts on different trophic levels in ecosystems (illustrated in Figure S1B).

In EcosystemModelInitialisation.csv, apart from the specification of the grid, the following parameters need to be set as follows:

- Length of simulation (years), 195

- Burn-in (years), 0

- Impact duration (years), 95

- Recovery duration (years), 100

- Read State,ReadModelState.csv

- Output Model State Timesteps,no

In Scenarios.csv, there should be a single line as follows:

label | npp | temperature | harvesting | simulation number

--- | --- | --- | --- | ---

NPPTED1 | temp-escalating-declining 0.01 0 | no 0.0 | no 0.0 | 10

This setup runs the escalation of vegetation removal up to 95% of NPP. To run lower simulations, the duration of impact needs to be reduced accordingly (for example, to 80 years for 80% eventual removal of NPP).

### Outputs

The outputs analysed in the manuscript are all given in the files prefixed 'GridOutputs'. There will be one produced for each replicate simulation (a total of 10 for the specification of simulations given above).

Biomass densities are transformed as log<sub>e</sub>(BD + 1), where BD is biomass density in units g km<sup>-2</sup>. Similarly, abundance densities are transformed as log<sub>e</sub>(AD + 1), where AD is abundance density in units individuals km<sup>-2</sup>. The calculation of mean trophic level is given in the Methods section of the manuscript. The calculation of the ranges of body masses and trophic levels present in an ecosystem was based on the variables Min Bodymass, Max Bodymass, Min Trophic Index and Max Trophic Index; this calculation is also described in the Methods section of the manuscript.
